# CASE REPORT Treatment of Otophyma: Case Report and Review of the Literature

**Published:** 2013-04-03

**Authors:** K. S. Sharma, J. Pollock, S. Hasham, T. M. Brotherston

**Affiliations:** Department of Plastic and Reconstructive Surgery, Sheffield Teaching Hospitals, Sheffield, United Kingdom

## Abstract

**Objectives:** Otophyma is a rare condition that can present as the end stage of any chronic inflammatory disease affecting the ear such as rosacea, eczema, or otitis externa. It can result in conductive hearing loss, low self-esteem, and social embarrassment. This report highlights a case of otophyma treated successfully using a full-thickness skin graft. **Methods:** We present a case of a 41-year-old lady referred to our department with a 23-year history of bilateral otophyma. During this time, her hearing progressively diminished as the swelling occluded her external auditory meatus. She had been unsuccessfully managed for years with topical emollients, steroids, and regular ear toileting. **Result:** She was treated by excision of the phymatous tissue and full-thickness grafting, which resulted in a patent external auditory meatus and an improvement in her hearing. **Conclusions:** The use of a full-thickness skin graft is one of the many treatment options available for the treatment of otophyma. We present a literature review on this uncommon condition and a discussion on the various treatment options available to the patient.

The phymas are defined as localized swellings of facial soft tissues due to variable combinations of fibrosis, sebaceous hyperplasia, and lymphoedema.^1^ The commonest is rhinophyma; however, other areas can be affected including the forehead (metrophyma), chin (gnathophyma), eyelids (blepharophyma), and ears (otophyma). Bilateral otophyma is a rare subtype of the family of phymas, with only a few cases described in world literature. This article presents a case with a 23-year history bilateral otophyma resulting in conductive hearing loss. Its assessment, investigations, pathology, surgical treatment, and a concise review of the literature are discussed.

## METHODS

A 41-year-old fit and well smoker was referred to our unit with a 23-year history of bilateral swelling of the conchal fossae secondary to relapsing, remitting otitis externa. Her initial presentation was typical of otitis externa, with painful, itchy ears and a persistent discharge. This was managed in the local ENT (Ear, Nose, Throat) department with regular aural toilet, topical and oral antibiotics, and topical steroid creams and drops.

## RESULTS

Despite this, her ears became progressively hypertrophied and skin excoriation of the pinna worsened, which prompted referral to the Dermatology department for further advice and management. Additional topical treatments were instigated but failed to improve the situation, and a diagnosis of severe eczematous otitis externa was reached. As the patient's ears began to hypertrophy further, she developed a 10- to 20-dB bilateral conductive deafness secondary to occlusion of the external auditory meatus as well as significant social embarrassment regarding their appearance. Because of the failure of management thus far, she was discussed in the local skin multidisciplinary meeting and referred to our Plastic Surgery unit for advice regarding surgical options to improve their appearance and improving her hearing.

**Figure 1:**
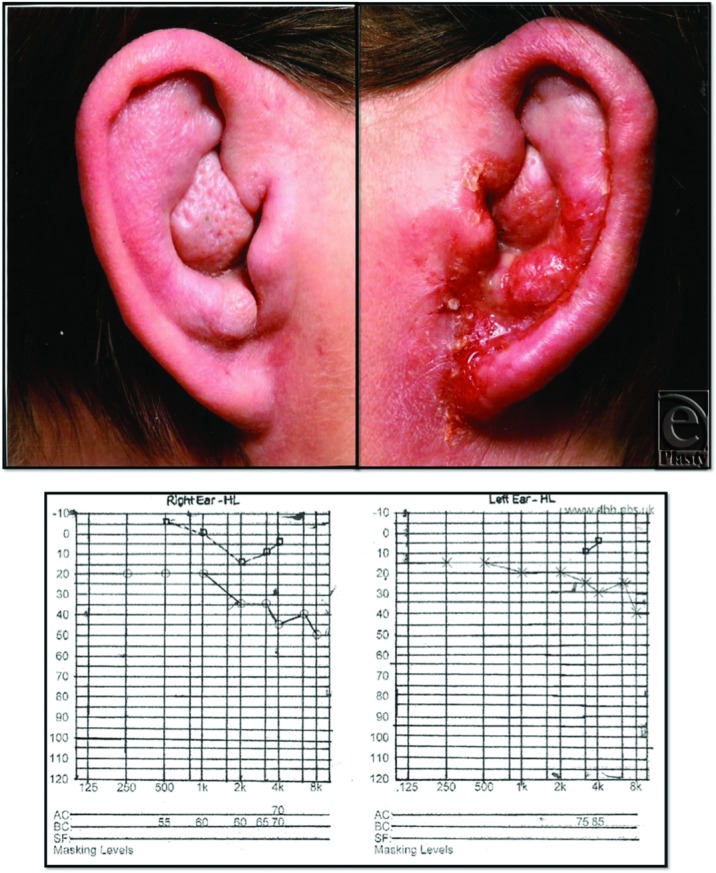
Images illustrating the preoperative appearance of bilateral otophyma secondary to chronic eczematous otitis externa with the right ear showing signs of infection (*top*). Preoperative pure tone audiograms illustrating conductive deafness worse on right compared to the left (*bottom*).

Examination of both ears revealed diffuse enlargement characterized by skin thickening and edema associated with accentuated adnexal pores and hair follicle ostia (peau d’orange appearance) affecting mainly the conchal fossa and antihelix. The external auditory canal was occluded by the papillomatous outgrowth in both ears preventing examination of the proximal canal and tympanic membrane. No signs of telangiectasia, papules, or extension of the swelling into the face or scalp were present. The left ear was excoriated and contained a mucopurulent discharge (Fig 1). There were no palpable lymph nodes in the head and neck. All routine blood and urine tests were within normal limits. Preoperative pure tune audiometry demonstrated conductive deafness of 15 to 20 dB in the right and 10 to 15 dB in the left ear (Fig 1). Magnetic resonance imaging and computed tomographic scans revealed no involvement of the underlying cartilages of both ears.

## DISCUSSION

After discussion of the options with the patient, a decision was made to excise the lymphedematous skin and resurface the resulting defect with a full-thickness skin graft, initially on the right ear, then the left at a later date when the discharge and excoriation settled. The aim of this was to create a patent external auditory meatus to improve the patient's hearing and allow egress of debris. Intraoperatively, the plane between the skin and the conchal cartilage was disrupted by coarse fibrous tissue resulting from the chronic inflammation. As a result, the affected cartilage was excised along with the overlying diseased skin. A full-thickness skin graft from the supraclavicular fossa was inset onto the deep surface of the posterior skin bridge and the remaining exposed perichondrium.

**Figure 2:**
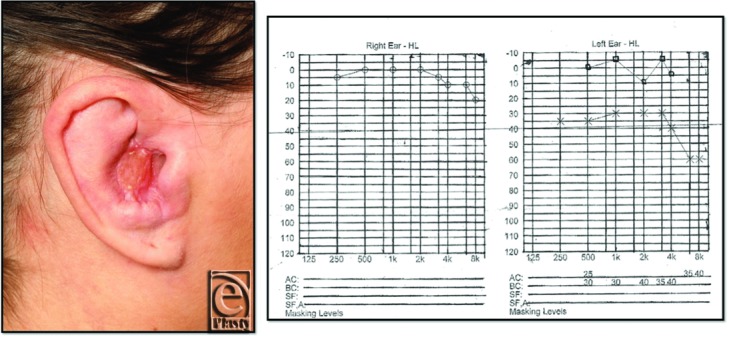
Postoperative images illustrating graft take on right ear 2 weeks postprocedure (*left*). Postoperative pure tone audiogram illustrating improvement in conductive deafness of the right ear (*right*).

Histology reported numerous plugged and dilated hair follicles with occasional epidermal cyst formation. Scarring, chronic inflammation, and focal edema were also noted. First graft check revealed some superficial epidermal sloughing, but the underlying dermis was pink and healthy. This was managed conservatively with dressings until healing occurred with in 3 weeks (Fig 2). Postoperative audiogram revealed thresholds within the normal range of hearing of the affected ear (Fig 2). An outpatient clinic visit at 8 weeks revealed healthy graft take with a patent external auditory meatus.

The phymas usually develop as a complication or end stage of rosacea but can result from inflammatory conditions such as eczema, psoriasis, atopic dermatitis; infections like erysipelas and cellulitis; trauma and primary or congenital lymphoedema.^2^^,^^3^ The initial stage is pitting which is reversible; followed by fibrosclerosis leading to irreversible, nonpitting, and hard edema associated with prominent follicular and adnexal ostia giving the classic peau d’orange appearance of the skin.^3^

Otophyma can be unilateral or more commonly bilateral. It tends to affect men more than women ranging from 4 to 66 years of age. It is a clinical diagnosis; however, biopsy aids in differentiation from other conditions with similar presentations such as sarcoidosis, carcinoma (basal, squamous, or sebaceous), and angiosarcoma.^4^ Histology of these phymatous tissues reveal hyperplasia and hypertrophy of the sebaceous glands with elongated, dilated, and plugged ducts. Irregular fibrous tissue proliferation is often present with a chronic inflammatory cell infiltrate and Demodex folliculorum infestation, a mite that can inhabit the pilosebaceous unit resulting in a foreign body–like reaction may be observed.^5^

Medical management is often instituted in an attempt to control the underlying disease process. In this case, the ENT and Dermatology specialists managed the patient for a number of years with a variety of oral and topical therapies. This intermittently controlled her symptoms of excoriation and discharge; however, it did not improve the appearance of her hypertrophic ears or hearing. Otophyma resulting from rosacea has been reported to improve using a combination of oral antimicrobials such as tetracycline, doxycycline, omidazole or metronidazole (used to treat D. Folliculorum) and retinoids like isotretinoin.^3^^,^^6^^,^^7^ Nevertheless spontaneous regression of this tumor like lesion is rare hence surgery and debulking remains the mainstay of long-term management.

The surgical treatment options of otophyma would seem to be best treated by mimicking that of rhinophyma.^8^ In the treatment of rhinophyma, the main aim is to remove the hypertrophied tissue and allow the defect to heal by secondary intention. Immediate or delayed reconstruction can be performed using a split-thickness or more commonly a full-thickness skin graft. While some of the rhinophyma treatment options have been described for otophyma—for example, of the use of local flaps to close the defect successfully reported in one case^5^—skin grafting has not been previously reported and was felt by the senior author to be the best method to provide an acceptable cosmesis and reduce the external acoustic meatus blockage leading to a conductive deafness. In our case, immediate resurfacing using a full-thickness skin graft was performed, which is a reliable method that provides a good texture and color match and the option of a local flap was still available if needed.

Decortication or partial excision is another technique that can be employed in the treatment of the phymas. The diseased tissue is shaved off in layers up to 2- to 3-mm above the underlying cartilage. Reepithelization depends on the proliferation of the remaining sebaceous glands, which can give a near normal texture and color match.^5^ The methods of decortication include cryosurgery, electrosurgery, chemical peels, dermabrasion, and the CO_2_ laser. The CO_2_ laser has become increasingly popular in the treatment of the phymas. It offers accuracy, hemostasis with better view of the surgical field and precise control of the depth and extent of vaporization.^8^ This can be done on an outpatient basis under local anesthesia. However, because of the long operative time, expense, and specialist training required, it is not widely available.

Otophyma can result in significant disfigurement of the ear and conductive deafness leading to psychosocial problems and reduced quality of life for those affected. Medical therapy has not proven to be satisfactory in management of the phymas and surgery remains one of the treatment options that can offer long-term benefit. This is the first case reported in the English language literature that describes successful use of a full-thickness skin graft as a reliable, easy, and convenient method of resurfacing these defects, with acceptable cosmetic and functional outcomes.
